# Diffuse Large B-cell Lymphoma of the Orbit With Intracranial Extension: A Rare Entity

**DOI:** 10.7759/cureus.47130

**Published:** 2023-10-16

**Authors:** Christopher Marsalisi, Hui Jun Guo, John M Sousou, Matthew Carpenter, Ahmad Alkhasawneh, Pramod Reddy

**Affiliations:** 1 Internal Medicine, University of Florida College of Medicine – Jacksonville, Jacksonville, USA; 2 Pathology, University of Florida Health – Jacksonville, Jacksonville, USA; 3 Pathology, University of Florida College of Medicine – Jacksonville, Jacksonville, USA

**Keywords:** r-chop chemotherapy, orbital malignancy, peri-orbital cellulitis, lymphoma, primary extranodal diffuse large b-cell lymphoma

## Abstract

Primary diffuse large B-cell lymphoma of the orbit is a rare diagnosis that accounts for less than 1% of all non-Hodgkin's lymphoma (NHL) cases. We present here the case of a middle-aged woman with a past medical history of intellectual delay and hypothyroidism who presented with a large diffusely infiltrating mass of the left orbit. A biopsy of the lesion during the patient's hospitalization confirmed a diagnosis of diffuse, large B-cell lymphoma. Due to extensive local invasion, she was deemed a poor surgical candidate. While inpatient, she was started on systemic chemotherapy and discharged with close follow-up planned with the oncologic and surgical teams.

## Introduction

Primary orbital lymphoma (POL) is a rare type of non-Hodgkin lymphoma that originates in the orbit or surrounding tissues. Non-Hodgkin's lymphoma (NHL) is a cancer that begins in the lymphatic system and commonly involves the lymph nodes; however, in about 40% of individuals, the primary lesion involves extranodal sites. Extranodal NHL typically presents as a constellation of primary organ pathologies with the most common of these sites being the gastrointestinal tract followed by the integumentary system and soft tissues [1].

When considering the broad classification of NHL, diffuse large B-cell lymphoma (DLBCL) is the most common. As with other lymphomas, DLBCL has a predilection for the lymphatic system with the primary lesions of the orbit being exceptionally rare, accounting for less than 1% of all NHL cases [2]. Orbital diffuse large B-cell lymphomas are characterized by their poor outcomes especially in the advanced stage (IV) and typically present as unilateral or bilateral, painless, indolent lesions [3]. Given the exceptional uniqueness of orbital, diffuse large B-cell lymphomas, we describe the case of a female with mental disabilities who presented to the hospital, accompanied by her mother, for swelling of the left eye. Biopsy confirmed the diagnosis of diffuse large B-cell lymphoma.

## Case presentation

A 56-year-old female with a past medical history of intellectual delay, mutism, and hypothyroidism presented to the emergency department (ED) with her mother for evaluation of left eye swelling. The patient was unable to communicate; therefore, medical history was obtained from the patient’s mother. The patient’s mother stated that for one year, she noticed gradual enlargement of what initially began as a small nodule over the medial aspect of the patient’s left eye. The patient’s mother stated that the lesion had recently started draining purulent fluid, which prompted her to bring the patient to the ED.

**Figure 1:**
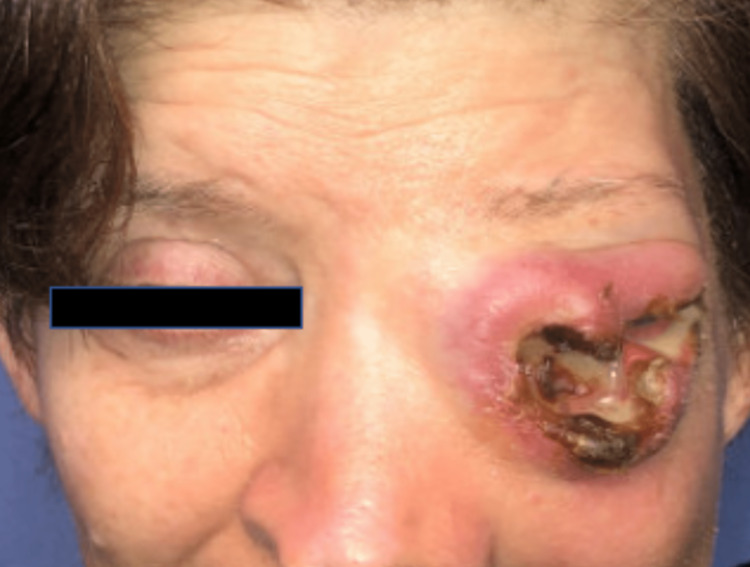
Physical exam of the patient's left orbit Physical exam findings demonstrating an erosive, ulcerating mass to the left eye with overlying erythema, edema excoriations, and purulence

In the ED, the patient’s vital signs were within normal limits and the laboratory analysis was unremarkable. Physical exam at this time was significant for a nonverbal, middle-aged female with an erosive, ulcerating mass to the left eye that had overlying erythema, edema, excoriations, and purulence (Figure 1). The ophthalmologic exam of the left eye was significant for corneal injection, cornea thinning nasally, three well-defined areas of corneal staining in the periphery, no light perception, and a narrow anterior chamber.

**Figure 2:**
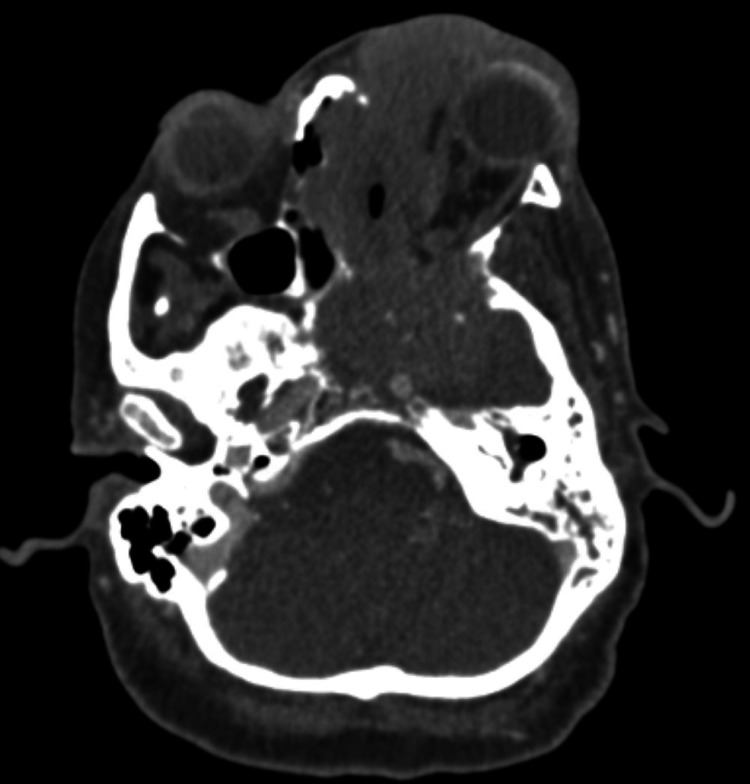
CT head with contrast A large diffusely infiltrating mass of orbital origin with prominent infiltration to the left maxillary sinus, left masticator space, left sphenoid bone, extending into the nasopharynx and invading the left nasopharyngeal soft tissues, nasal cavity, bilateral ethmoid air cells, and bilateral sphenoid sinuses. The mass extends anteriorly in the left nasal cavity and through the left face into the medial canthus region with an ulcerated aspect along the left periorbital soft tissues. There is an associated displacement of the left globe with the proptotic appearance of the left globe from mass effect with the encroachment of the mass near the fat plane along the left medial rectus.

Computed tomography (CT) of the head in the ED demonstrated an infiltrative mass lesion originating in the orbit, measuring 8.7 cm x 6.0 cm x 9.0 cm with diffuse bony erosion of the walls of the left maxillary sinus through the left nasal cavity into the bilateral ethmoid air cells and the bilateral sphenoid sinus. The lesion was noted to be invading and eroding the adjacent skull base as well as the left carotid canal and the cribriform plate (Figure 2). Out of concern for an orbital neoplasm with superimposed infection, the patient was started on vancomycin and admitted to the hospital for further workup and management.

**Figure 3:**
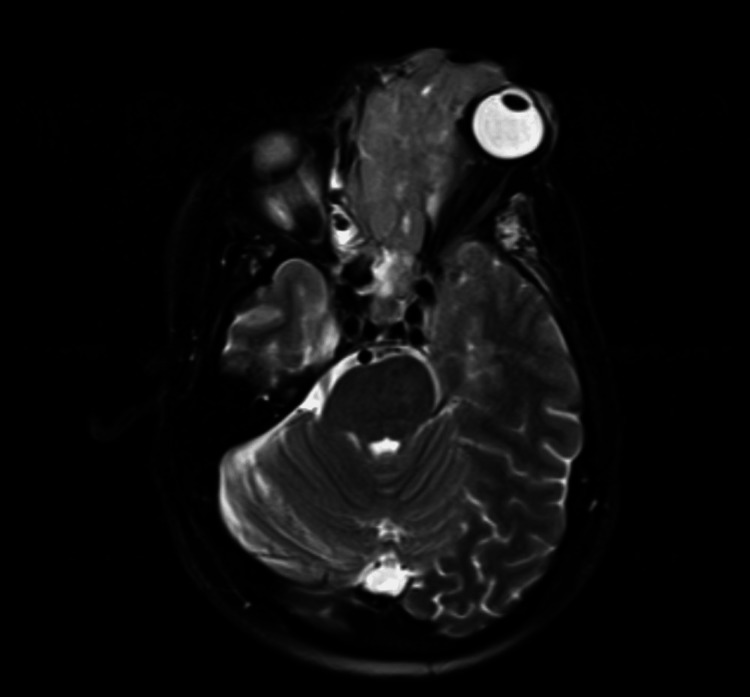
MRI brain with contrast MRI brain, transverse plane of infiltrative mass lesion originating in the orbit measuring with erosion of the walls of the left maxillary sinus, through the left nasal cavity into the bilateral ethmoid air cells, and bilateral sphenoid sinus. This view demonstrates the erosion of the adjacent skull base as well as the left carotid canal and the cribriform plate.

**Figure 4:**
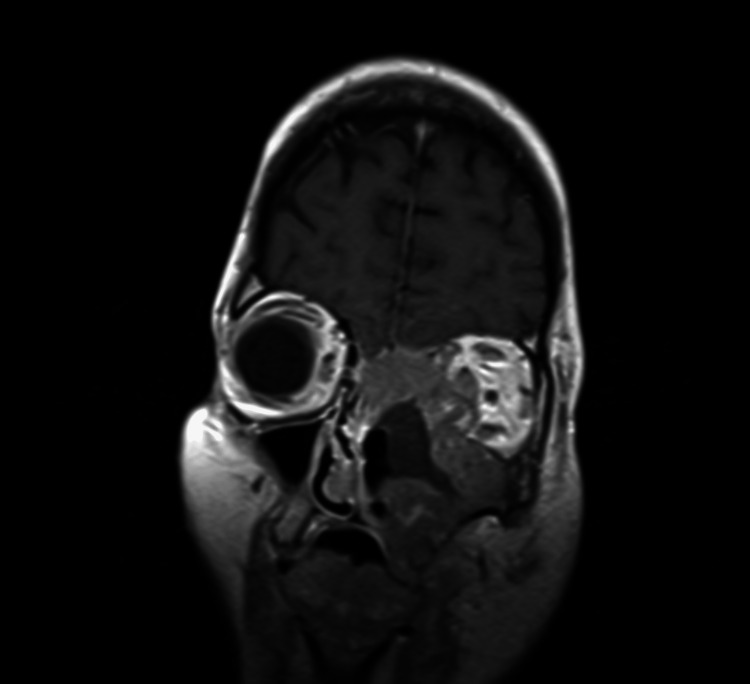
MRI brain with contrast MRI brain, coronal view of the left orbital lesion extending into the left sinonasal cavity and left preseptal tissues, nasopharynx, and left pharyngeal and masticator spaces.

**Figure 5:**
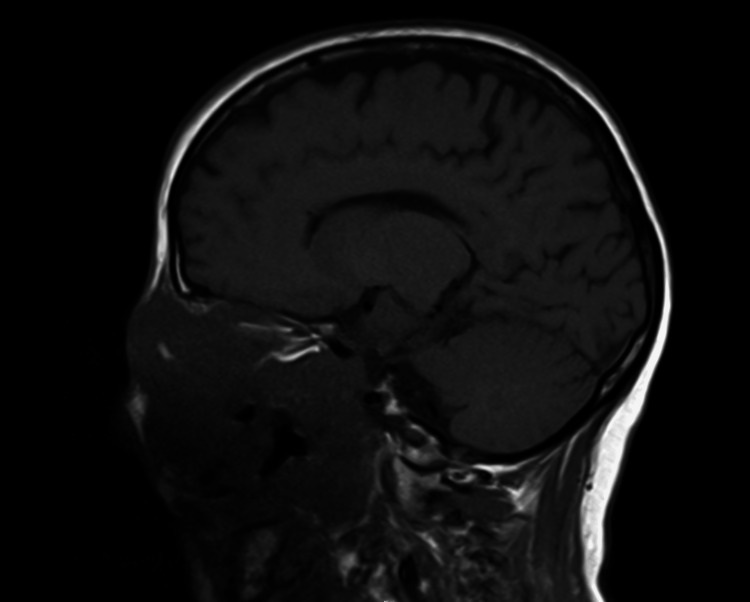
MRI brain with contrast MRI brain, sagittal plane, demonstrating the epidural expansion of the neoplasm along the floor of the anterior cranial fossa (left>right) with the abutment of the bilateral inferior frontal lobes. This view also allows for visualization of the epidural expansion into the left middle cranial fossa inseparable from the underlying anterior left frontal lobe.

Upon admission, the ophthalmology, oral and maxillofacial surgery, neurosurgery, and oncological teams were consulted and further imaging was obtained. MRI of the brain detailed epidural expansion of the lesion along the floor of the anterior cranial fossa (left more than right) with abutment of bilateral inferior frontal lobes. There was also epidural extension into the left middle cranial fossa inseparable from the underlying anterior frontal lobe (Figures 3-5).

**Figure 6:**
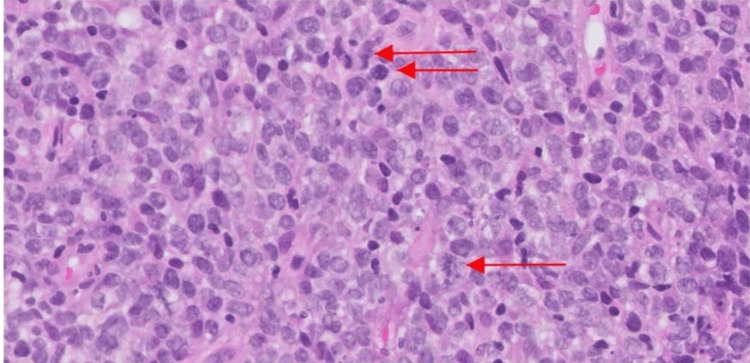
Pathology results from tissue biopsy Pathology from tissue biopsy demonstrating CD20 immunoreactive lymphocytes (red arrows) supporting a diagnosis of diffuse large B-cell lymphoma

Consulting teams recommended a punch biopsy of the lesion, which resulted in an atypical lymphoid infiltrate consistent with diffuse large B-cell lymphoma (DLBCL) (CD20: positive) (Figure 6). Fluorescence in situ hybridization (FISH) revealed loss of the BCL6 region on chromosome 3 and loss of chromosome 8, including its MYC gene region. FISH was negative for Bcl6 rearrangement, MYC rearrangement, MYC amplification, BCL2 rearrangement, and t(8;14). In an effort to stage the malignancy, a CT of the chest, abdomen, and pelvis was ordered. Results were significant for clustered nodules throughout the right lung, a left supraclavicular lymph node, retroperitoneal lymphadenopathy, and an enlarged para-aortic lymph node. At this time, the malignancy was classified as stage III diffuse B-cell lymphoma.

Due to the expansion of the DLBCL into the epidural space, a lumbar puncture was performed, which resulted in an unremarkable CSF fluid analysis. A bone marrow biopsy performed during hospitalization had unremarkable flow cytometry and surgical pathology. Mycobacterial cultures of the lesion grew *Staphylococcus *aureus, and the patient was committed to six weeks of IV antibiotics.

The extensive invasion of the patient's malignancy into her cranium made her a poor surgical candidate and, after an extensive multidisciplinary discussion, a decision was made to offer the patient chemotherapy. The patient's mother, the healthcare surrogate, was amenable to the plan, and the patient was started on a combination of cyclophosphamide doxorubicin, vincristine, solumedrol, and rituximab (R-CHOP). The patient tolerated two cycles of chemotherapy well while inpatient and was discharged with plans to follow up with oncology and ophthalmology.

Unfortunately, according to hospital records, the patient missed multiple follow-up appointments and was reported to have passed away two months after the presented hospitalization. Outside medical records were obtained and did not detail any recent admissions or visits with providers at other institutions.

## Discussion

Primary orbital lymphomas are a diverse group of tumors that are commonly characterized by their low-grade classification, with only about 16% being high-grade lymphomas. The most common type of primary orbital lymphomas are mucosa-associated lymphoid tissue (MALT) followed by follicular lymphomas, diffuse large B-cell lymphomas (DLCL), and mantle cell lymphomas [4]. When considering the progression of primary orbital lymphomas, the malignancy is characterized by an indolent course that is typically slowly growing and commonly asymptomatic. Occasionally these tumors are associated with proptosis, periorbital swelling, ocular dysmotility, and blurring of vision [4]. In the reported case, the patient presented with advanced malignancy and extensive invasion into the calvarium.

The diagnosis of orbital lymphomas may be challenging, as the malignancy does not present with classic findings. On computerized tomography, these malignancies are typically characterized by a heterogeneous soft tissue density, which may have areas of calcifications and/or phleboliths. Advanced imaging with magnetic resonance commonly demonstrates an isointense T1 signal, bright T2 signal, and dark internal septations [5]. Although imaging studies may provide important information regarding the identification and progression of orbital lymphomas, the gold standard for diagnosis is an orbital biopsy with a histological diagnosis.

In the presented case, histological analysis of the orbital lymphoma resulted in DLBCL (CD20: positive). This POL subtype is one of the most commonly diagnosed and is associated with the most outcomes. A large cohort study, published in 2022, focused on determining the prognostic characteristics of DBCL. These researchers discovered that older age at the time of diagnosis and stage IV malignancies were two main characteristics that confer the worst outcomes [2]. Furthermore, the results from this study predicted shorter overall survival time in patients older than 75 years old (hazard ratio of 2.324 and p-value: 0.007). In the presented case, the patient would have been expected to have a better prognosis based on her age (hazard ratio for overall survival of 4.8888 with a p-value of 0.0001) and tumor stage. Despite these convincing data points, the patient's course, and eventual demise are attributed to the extensive invasion and destruction of the central nervous system architecture.

In a recent multicenter study evaluating treatment modalities in DLBCL POL, and resultant outcomes, it was found that there is notable intracenter and intercenter variability. Despite this, the most commonly utilized treatment modality was reported to be external beam radiation therapy (EBRT) followed by R-CHOP [6]. In this study, the five-year overall survival rate of the entire cohort was 36.0%, with relapses noted in 43.9%. In the presented case, the patient was started on systemic chemotherapy after treatment options were discussed among specialists and presented to the patient’s mother.

## Conclusions

Diffuse large B-cell lymphoma is one of the most prevalent types of primary orbital lymphoma with limited case reports detailing this malignancy’s potential for local invasion into the central nervous system. This case emphasizes multiple diagnostic challenges of orbital lymphomas and why this may delay early diagnoses. In the presented case, as with many other cases of orbital lymphomas, the malignancy was not recognized promptly due to nonspecific symptoms. Another teaching point that this case reinforces is the importance of providing medical care for individuals with intellectual or developmental disabilities. This care requires a comprehensive and patient-centered approach tailored to the patient’s specific needs and circumstances in collaboration with the primary caregiver. We hope, through the presentation of this case, to emphasize this rare entity and the serious complications that can result.
